# SNP discovery and association mapping in Eucalyptus pilularis (blackbutt)

**DOI:** 10.1186/1753-6561-5-S7-O9

**Published:** 2011-09-13

**Authors:** Timothy Sexton, Robert Henry, Chris Harwood, Dane Thomas, Luke McManus, Carolyn Raymond, Michael Henson, Mervyn Shepherd

**Affiliations:** 1 CRC for Forestry, Centre for Plant Conservation Genetics, Southern Cross University, Lismore, New South Wales, 2480, Australia; 2 Queensland Alliance for Agriculture and Food Innovation, The University of Queensland Brisbane, Queensland, 4072, Australia; 3 CSIRO Ecosystem Sciences, Private Bag 12, Hobart, Tasmania, 7001, Australia; 4 South Australian Research and Development Institute, Wine Innovation Central Building, Waite Research Precinct, corner of Hartley Grove and Paratoo Road, Urrbrae, 5063, Australia; 5 CRC for Forestry, Forest Molecular Biology and Genetics Group, The University of Melbourne, Creswick, Victoria, 3363, Australia; 6 Centre for Plant Conservation Genetics, Southern Cross University, Lismore, New South Wales, 2480, Australia; 7 Eucalyptus.biz Limited, Rosemead, Polesden Lane, Ripley, Surrey, GU23 6DX, UK

## Background

This research explores the universality of genetic variation in genes controlling wood formation across the genus *Eucalyptus*.

Breeding and deploying Eucalypts for improved wood quality is constrained by the delay before wood traits can be measured reliably. Marker assisted selection (MAS) offers a way to make earlier selection of wood properties, by selecting Single Nucleotide Polymorphisms (SNPs) in the DNA which can predict specific phenotypic traits (genotype-phenotype links). SNPs shared between species (trans-specific SNPs) may have broad application to multiple species. When the species are more distantly related, ancient SNPs shared between subgenera (trans-subgeneric SNPs) are likely to be of adaptive importance and persist in separate lineages due to balancing selection [1].

## Sample and methods

The focal species of this investigation was *Eucalyptus pilularis* of the subgenus *Monocalyptus*, one of the most important species for solid wood production in Australia. As part of the development of suitable SNP markers for *E. pilularis*, two other important species were included for comparative purposes. The first comparison was made with the closely related *Eucalyptus pyrocarpa*, which grows in parapatry with *E. pilularis* and is distinguished by minor morphological characteristics including a larger leaf and capsule size [2]. More distant comparisons were made between both *Monocalyptus* species and the alternate *Symphyomyrtus* subgenus, represented by *Eucalyptus globulus* subspecies *globulus*.

DNA was pooled from 30 individuals representing the natural geographical range of each of three species. A total of 34 genes were amplified from each pool and sequenced on an Illumina GAIIx. SNPs were identified in individual species pools and classified as trans-specific if they shared the same reference position in two or more species.

The association study focused on *E. pilularis* and utilized 561 destructively sampled trees from a nine-year-old progeny trial established by Forests NSW at Hannam Vale, near Port Macquarie in New South Wales, Australia (Latitude 31°40’, Longitude 152°33). This sample represented the genetic diversity of 284 open-pollinated families, collected across 37 provenances that encompassed a large part of the natural distribution of this species. We tested 40 traits covering growth, wood chemistry (lignin, cellulose, hemi-cellulose complexes), and wood dimensional stability, strength and stiffness [3]. Eight SNPs were genotyped in pectin methylesterase 6 and 7 (*PME6* and *7*) using Sequenom iPLEX gold chemistry. Association testing was performed using a General Linear Model (GLM) in TASSEL where 1,000 permutations were applied. Kinship between family members was a problem and was addressed by the inclusion of a kinship matrix as covariate data within the model. The large population size used also allowed for validation of results in smaller subsets of the population containing only single representatives from each family. Previous studies have reported no geographical population structure for the provenances included in the breeding program [4].

## Results and discussion

Among the three species 6,852 SNPs were identified in total. Pairwise comparisons between species representative of divergent subgenera revealed that 20-23% of SNPs were shared. These trans-subgeneric SNPs are likely to have persisted in separate lineages for tens of millions of years following the split of the *Monocalyptus* and *Symphyomyrtus* subgenera and may be indicative of adaptive variation maintained by “balancing selection”. The primary signatures of balancing selection were significantly higher proportions of trans-subgeneric SNPs in exons and promoters, compared to introns and 3’ untranslated regions. Further analysis revealed that ten of the 34 genes investigated were likely to be influenced by balancing selection with a significantly higher proportion of trans-subgeneric SNPs and high nucleotide diversities.

*PME6* and *PME7* were two of the genes considered likely to be influenced by balancing selection. Association testing revealed several significant correlations between SNPs in these genes and solid wood properties of *E. pilularis*. *PME6* was primarily associated with shrinkage of drying timber. This is consistent with a role for pectin as a hydrophilic polysaccharide, and patterns of methyl-esterification are known to affect the water holding capacity of pectin gels. *PME7* was primarily associated with cellulose and pulp yield, and inversely with lignin content. Selection of specific alleles in these genes may enable identification of trees with superior wood quality for breeding and deployment. For some SNPs, the heterozygote was superior, indicating that selection to fix one allele in the breeding population would not be appropriate. In combination with haplotype blocks, a heterozygote advantage at one or more *PME7* loci may explain the absence of one homozygous class at all SNPs investigated in this gene. Alternatively the absent homozygous genotypes may be disadvantaged during early life stages.

## Conclusions

Using comparative genomics we provided direct DNA-based evidence that many candidate genes for wood formation are under balancing selection and therefore of adaptive importance. Such knowledge of genetic variation maintained by balancing selection could be critical to ensure that selection for alleles linked to desirable phenotypes does not compromise the maintenance of adaptability in the breeding population. This concept needs to be tested, and our experiments provide a number of suitable markers which could provide the basis for further investigation.

Each of these informative SNPs in both *PME* genes could be used for MAS, subject to cost-benefit analysis. Given the large number of trans-subgeneric SNPs identified in these genes, many informative SNPs are likely to transfer to other species, facilitating the development of SNP markers in other commercial species within the diverse *Eucalyptus* genus.
